# Novel Molecular Targets for the Treatment of Gastroenteropancreatic Endocrine Tumors: Answers and Unsolved Problems

**DOI:** 10.3390/ijms14010030

**Published:** 2012-12-20

**Authors:** Gabriele Capurso, Volker Fendrich, Maria Rinzivillo, Francesco Panzuto, Detlef K. Bartsch, Gianfranco Delle Fave

**Affiliations:** 1 Digestive and Liver Disease Unit, S. Andrea Hospital, Faculty of Medicine and Psychology, Sapienza University of Rome at S. Andrea Hospital, Via di Grottarossa 1035, 00189 Rome, Italy; E-Mails: gabriele.capurso@gmail.com (G.C.); mariarinzivillo@gmail.com (M.R.); francesco.panzuto@gmail.com (F.P.); 2 Department of Surgery, Philipps-University, Marburg, Germany; E-Mails: fendrich@med.uni-marburg.de (V.F.); bartsch@med.uni-marburg.de (D.K.B.)

**Keywords:** gastroenteropancreatic endocrine tumors, target therapy, mTOR, angiogenesis, Src, hedgehog, combined treatment, resistance

## Abstract

As more knowledge on molecular alterations favoring carcinogenesis and spreading of gastroenteropancreatic endocrine tumors has become available, a number of targeted agents interfering with key growth and angiogenic pathways have been explored in preclinical and clinical studies. The mTOR inhibitor Everolimus, and the multi-target antiangiogenetic agent Sunitinib, have been shown to be effective and thus have been approved by the FDA for treatment of pancreatic endocrine tumors. However, there is little data on the primary resistance to targeted agents on these tumors. The goals of the present review are to elucidate the possible advantage of combined treatments in overcoming induced resistances, and to identify biomarkers able to predict clinical efficacy. Moreover, the role of interesting targets for which a strong biological rationale exists, and specific inhibitors are available, such as the Src Family Kinases and the Hedgehog Pathway, are discussed. There is now need for more preclinical studies on cell lines and animal models to provide a stronger preclinical background in this field, as well as clinical trials specifically comparing one targeted therapy with another or combining different targeted agents.

## 1. Introduction

Gastroenteropancreatic endocrine tumors (GEP ETs) represent a heterogeneous group of neoplasms deriving from the gastrointestinal (GI) tract and pancreas diffuse neuroendocrine system [1].

Although considered rare entities, their incidence seems to be increasing by up to five cases per 100,000 persons per year [2]. In addition, due to long-term survival, their prevalence is even higher than those of oesophageal, gastric, pancreatic and hepatobiliary cancers in the US [2].

GEP ETs are generally considered “indolent” tumors, but two thirds of them present with metastatic diseases, and are often not suitable for radical surgery [3,4]. Prognosis of GEP-ETs depends on a number of variables, including primary tumor site, disease staging at time of diagnosis, and tumor proliferative activity, which is mainly expressed by the Ki67 value on tumor cells [5].

As far as medical treatment is concerned, several options have been proposed in the past, including somatostatin analogues [6], peptide receptors radionuclide therapy (PRRT) [7], and systemic chemotherapy [8]. Despite these therapeutic tools, the majority of advanced GEP-ETs have a progressive behaviour, particularly in those cases with higher Ki67 value [9,10].

In the past few years, as more knowledge on molecular alterations favouring carcinogenesis and spreading of GEP ETs has become available, a number of targeted agents interfering with key growth and angiogenic pathways have been explored in preclinical and clinical studies as novel and promising tools for GEP ETs treatment [11–13]. Recently, the mTOR inhibitor, Everolimus, and the multi-target antiangiogenetic agent, Sunitinib, have been shown to be effective in prolonging progression-free survival (PFS) in advanced progressive pancreatic endocrine tumors (PETs), and thus have been approved by the FDA for treatment of this disease [14].

However, a number of other compounds are under investigation. The present review will summarize existing data on targeted therapies for GEP ETs, focusing on combined treatments and novel targeted agents.

## 2. Targeting Angiogenesis

The inhibition of neoangiogenesis is considered a valid treatment approach, achieving good results in a number of solid tumors [15]. Angiogenesis is a central and complex process in tumor growth and metastasis, and involves a number of receptor tyrosine kinases (RTKs) and their ligands. Vascular endothelial growth factor (VEGF) is a specific key driver of angiogenesis in pancreatic endocrine tumors (PETs) [11].

The investigation of the VEGF pathway is also of particular interest for the biology of GEP ETs, especially of PETs, as angiogenesis switch, coupled by progressive expression of VEGF are key mechanisms in the transgenic mouse model (Rip1-Tag2) developing PETs [16]. The role of vascularisation in PETs is somewhat controversial, with some studies reporting that expression of VEGF correlates with a more aggressive tumor behaviour [17,18], and others that malignant tumors show lower VEGF expression than benign ones [19].

As tissues from malignant PETs also shows widespread expression of VEGF receptors (VEGFR), platelet-derived growth factor receptors (PDGFRs) α and β, and other receptors such as the stem-cell factor receptor (c-kit) [20], inhibitors of these kinases, have been tested in GEP ETs patients.

### 2.1. Sunitinib

Sunitinib (Sutent^®^, Pfizer) is a multitargeted tyrosine kinase inhibitor with an antiproliferative and antiangiogenic effect, with activity against VEGFR, PDGFR, c-KIT, Flt-3 and RET [21]. In a phase I trial, 28 patients with different types of cancer received between 50 mg and 150 mg/day of Sunitinib. Out of the four patients with GEP ETs, one had a partial response and a second achieved a minor response [22].

The efficacy and safety of Sunitinib have been assessed in a large phase II study of 109 patients with advanced, unresectable GEP ETs treated with repeated 6-week cycles of Sunitinib (50 mg/day, 4 weeks on and 2 weeks off). Radiological response was observed in 13.5% and 5.1% of PETs and carcinoid tumors, respectively, along with high percentages of SD and an acceptable safety profile [23]. Unfortunately, it has not been specified whether treatment showed efficacy in patients with PD at study entry. The median time to progression (TTP) was 10.2 months for patients with carcinoids and 7.7 for patients with PETs.

Sunitinib received approval in Europe and the US for the treatment of progressive, well-differentiated pancreatic neuroendocrine tumors in adult patients with unresectable, locally advanced or metastatic disease. The approval was based on a randomized controlled trial of Sunitinib, 37.5 mg daily (86 patients) *versus* placebo (85 patients). The primary endpoint was PFS as assessed by investigator assessment. The median PFS for Sunitinib was 10.2 months, compared with 5.4 months for placebo (HR 0.427, CI 95% 0.271, 0.673; *p* < 0.001). Overall survival was also improved, as nine deaths were reported in the Sunitinib group (10%) and 21 deaths were reported in the placebo group (25%), with a hazard ratio of 0.41 (95% CI, 0.19 to 0.89; *p* = 0.02) in favour of Sunitinib, suggesting a reduction of the risk of death of 59% at the intention to treat analysis [24].

Overall, up to 59% of patients receiving Sunitinib experienced side effects, which were mild grade 1–2 toxicity in most cases. However, severe grade 3–4 were observed in 12% and 10% of patients, respectively [24].

There are no clinical data on the use of Sunitinib in endocrine tumors other than PETs. However, a clinical trial aimed at evaluating the activity of Sunitinib, alone or in combination with the somatostatin analogue lanreotide, in midgut carcinoids has been endorsed by the European Neuroendocrine Tumour Society (ENETs) (SUNLAND: Sunitinib and LANreotide in carcinoiDs).

### 2.2. Other Angiogenesis Inhibitors

Initial clinical trials investigating anti-VEGF-related therapy included the use of Sorafenib (Nexavar^®^, Bayer Pharma AG) and Bevacizumab (Avastin^®^, Roche). Sorafenib is a multiple kinase inhibitor affecting the Vascular Endothelial Growth Factor Receptor 2 (VEGFR2), Platelet-derived growth factor receptor (PDGFR), Fibroblast growth factor receptor 1 (FGFR1) and FMS-like tyrosine kinase 3 (FLT3) [25]. Bevacizumab demonstrated antitumoral activity in a GEP ETs mouse model, where it inhibited tumor angiogenesis and impaired tumor growth [17]. Few clinical trials have been published to date, evaluating Bevacizumab use in GEP ETs. In a phase II study, Bevacizumab was shown to have modest activity in GEP ETs [26] with an objective response rate (ORR) of 7%–11% in non-selected GEP ETs.

In a phase II study, temozolomide and Bevacizumab were safely administered in combination, in patients with advanced GEP ETs, but the combination regimen appeared promising only for patients with pancreatic tumors [27]. Moreover, when Bevacizumab was administered alongside SSAs, as compared to IFN-a, PFS was improved in the Bevacizumab arm when compared to IFNa monotherapy [28].

Brivanib (Bristol-Myers Squibb) is a novel agent with dual-inhibitor activity of FGF and VEGF, which shows activity and improves survival in the RIP-Tag2 mouse model of PETs [29]. This agent is planned for further clinical evaluation. Pazopanib (Votrient, Glaxo Group Ltd.) is an orally available angiogenesis inhibitor that targets VEGFR1, -2 and -3; PDGFRα and c-kit. Current phase II trials include monotherapy in low-intermediate-grade PETs. Current trials of pazopanib in combination with other agents include temozolomide in PETs and Everolimus during embolisation with SIR spheres. Cabozantinib (XL184, Exelixis) is another novel small molecule kinase inhibitor that inhibits MET and VEGFR2 and has been shown to suppress metastasis, angiogenesis and tumor growth in early phase trials [30], an open-label phase II study in advanced GEP ETs is due to open shortly.

All the above mentioned results suggest that inhibition of angiogenesis can be an effective way to treat PETs. However, it should be kept in mind that: (a) the less vascularised and more aggressive tumors might respond less to the treatment. (b) that VEGFR inhibitors might induce hypoxia and induce the synthesis of other proangiogenic factors not responding to treatment, potentially leading to a more aggressive disease, as described in preclinical models [31]. (c) there are no solid data on the clinical usefulness of biomarkers able to predict which patients would respond better to such treatments. From this standpoint, it could be of more interest to investigate the role of the Von Hippel-Lindau (VHL) gene and of Hypoxia-inducible factors-1α (HIF-1α). Indeed, the rationale for treatment with anti-VEGF targeted therapies is supported by the high frequency of alterations of the VHL gene in PETs. VHL silencing would result in increased HIF-1α activity eventually driving tumor angiogenesis [32]. Thus, tumors with mutated VHL and increased HIF-1α might respond better to such drugs.

## 3. Targeting the PI3K-mTOR Pathway

The mammalian target of rapamycin (mTOR) is a highly conserved serine/threonine kinase that regulates cell cycle and metabolism in response to environmental factors. mTOR mediates signaling transduction downstream of receptor tyrosine kinases, thus playing a critical role in different proliferative signals mediated through the phosphatidylinositol 3 kinase (PI3K)/protein kinase B (AKT) pathway. The signaling pathways upstream of mTOR include several tumor suppressor genes, such as Phosphatase and tensin homolog (PTEN), Neurofibromatosis1 (NF1), and the tuberous sclerosis complex (TSC1/TSC2), which negatively regulate mTOR.

A number of studies investigated the expression of genes belonging to the PI3K/AKT/mTOR pathway in GEP ETs, suggesting its activation, which seems mainly due to mutations or reduced expression of its negative regulators, such as PTEN and TSC2 [33,34]. The activation of the mTOR pathway in pancreatic endocrine tumors is also supported by immunohistochemical expression of p-mTOR [35], and its downstream effector eukaryotic translation initiation factor 4E binding protein 1 (p-4E-BP1) which has also been reported to be an independent factor of poor prognosis [36].

### 3.1. Everolimus

Everolimus (Afinitor, Novartis Oncology) is a potent, orally available inhibitor of mTOR. Preclinical studies demonstrated a constitutive activation of the PI3K/AKT/mTOR signalling pathway in GEP ETs cells, and showed that inhibition of mTOR by rapamycin or Everolimus is able to reduce cell growth [34,37,38].

On this basis, Everolimus has been tested in patients with GEP ETs. In a phase II study [39], two different doses of Everolimus (5 and 10 mg a day) combined with Octreotide LAR (Sandostatin^®^ LAR^®^, Novartis Oncology) was administered to 60 patients with GEP-ETs. Partial response (PR) was observed in 22% of these patients, whereas disease stabilization (SD) was reported in 70% of them. Treatment was well tolerated, severe G3-G4 side effects being reported in 10% of patients. The daily 10 mg dose showed better results compared with the 5 mg dosage.

In a subsequent phase II trial (RAD001 In Advanced Neuroendocrine Tumors: RADIANT-1 trial) the activity of Everolimus, 10 mg a day, was confirmed in 160 patients with progressive well differentiated PETs after failure of a previous chemotherapy [40]. Two strata were considered: stratum 1 for 115 patients receiving Everolimus alone, and stratum 2 with 45 patients receiving Everolimus and octreotide LAR. Interestingly, a clinical benefit (proportion of patients with partial response or disease stabilization) was observed in 77.4% and 84.4% of patients in stratum 1 and 2, respectively, thus suggesting a possible synergism of anti tumor action between Everolimus and octreotide. Severe grade 3–4 toxicity was confirmed to be present in some 5% of patients receiving Everolimus.

Everolimus received approval in 2011 for the treatment of patients with progressive, unresectable, locally advanced or metastatic neuroendocrine tumors of pancreatic origin (PETs), based on the results of the RADIANT 3 trial of Everolimus *vs.* placebo in patients with advanced progressive PETs [41]. To date, this is the largest available, prospective, randomized double blind study in GEP-ETs, evaluating 410 patients with advanced progressive low or intermediate-grade PETs. Progression-free survival was significantly improved in the 207 patients who received Everolimus, as compared with the 203 patients who received placebo, median PFS being 11 months and 4.6 months in the two groups of patients, respectively. Grade 3 or 4 events that were more frequent with Everolimus than with placebo included anemia (6% *vs.* 0%) and hyperglycaemia (5% *vs.* 2%). No difference in terms of overall survival was observed between patients receiving Everolimus or placebo. However, this difference might be related to the crossed-over design, with patients having progression on placebo being switched to Everolimus.

Few data are available concerning the efficacy of Everolimus in non-pancreatic endocrine tumors. The RADIANT 2 trial, a randomised double-blind placebo-controlled phase 3 study, compared 10 mg Everolimus plus octreotide LAR *vs.* placebo plus octeotide in 429 patients with well-differentiated advanced, progressive NETs associated with carcinoid syndrome, with various primary site. The median PFS was 16.4 months in the Everolimus plus octreotide LAR group and 11,3 months in the placebo plus octreotide LAR group (HR 0.77), thus resulting in a 23% reduction in the estimated risk of progression. Although the difference in terms of PFS between the two groups of patients was significant (*p* = 0.026), the adjusted pre-specified p value of 0.024 was not reached [42].

Two further ongoing clinical trials are assessing the efficacy of this drug in patients with a non pancreatic endocrine tumor, without a carcinoid syndrome (RADIANT4, NCT01524783 and RAMSETE, NCT00688623).

However, there are several issues that need to be addressed for an optimal clinical use of Everolimus or other mTOR inhibitors. In particular: (a) the capacity of tumor cells to develop escape pathways during treatments with mTOR inhibitors may suggest that combined treatments with other drugs may prove beneficial [43]. (b) the lack of biomarkers able to predict the response of patients to the treatment with Everolimus. In this view, recent data comparing the levels of pAKT on paired tumor biopsies obtained before and after treatment with Everolimus would suggest that an increase in p-Akt with treatment is more common in patients obtaining a better response to treatment [44], possibly suggesting that induced Akt phosphorylation is not necessary a marker of resistance to mTOR inhibitors, as previously suggested.

### 3.2. Novel Inhibitors of the PI3K-AKT-mTOR Pathway

As discussed above, a number of mechanisms can lead to primary or acquired resistance of cancer cells to mTOR inhibitors, including activation of alternative pathways, due to other mutations, and excessive reactivation of AKT feedback resulting in overactivation of the insulin-growth factor.

The rationale approaches to avoid these unwanted escapes include either a “horizontal blockage” of other pathways together with that of the mTOR [36,43], or a “vertical blockage” of the PI3K-AKT-mTOR pathway at different levels.

Therefore, a number of preclinical and clinical investigations have been aimed at evaluating the efficacy of mTOR kinase inhibitors that would inhibit both mTORC1 as well as mTORC2 (thus inhibiting Akt phosphorylation), or dual PI3K/mTOR inhibitors.

BEZ235 (Novartis Oncology) is a pan-class I PI3K inhibitor. This effect is mediated through binding to Valine-882 and Serine-805 in the hinge region of ATP-binding pocket of the p110 subunit of PI3K, displaying a slight preference for the alpha-isoform. In addition BEZ235 binds to the catalytic site of m-TOR, inhibiting both m-TOR complexes (mTORC1 and mTORC2).

BEZ235 down-regulates direct and indirect downstream effectors of PI3K such as AKT, GSK3Beta, p70S6K and ribosomal protein S6 in preclinical models and effectively inhibits tumor proliferation and growth in a variety of models including cells lines and xenografts. BEZ235 further demonstrated anti-angiogenic effects [45].

Other oral inhibitors of the PI3K pathways are BKM120 (Novartis Oncology) (pan-PI3K inhibitor) and BYL719 (Novartis Oncology) (selectively inhibiting PI3Kalpha) [46].

BEZ235 and BKM120 are currently being investigated in Phase I and II clinical trials in advanced solid tumor patients as a single agent or in combination with other antineoplastic therapy.

The dual mammalian target of rapamycin complex (mTORC) 1/mTORC2 inhibitor, AZD8055 has also already been shown to have a good tolerability in patients with solid tumors [47], without objective responses, although no GEP ETs were included in these studies.

Interestingly, in a recent preclinical study BEZ235 was more effective than rapamycin in inhibiting the cell proliferation and inducing apoptosis in a rat insulinoma cell line [48].

BEZ235 will soon be specifically investigated in a phase II trial of BEZ 235 compared with Everolimus, in patients with advanced PETs (NCT01658436).

## 4. Prospect for Src Inhibitors

The non-receptor protein tyrosine, Src, is the archetypal member of a family of nine membrane associated-tyrosine kinases (SFKs), the other members being: Lck, Fyn, Yes, Hck, Blk, Fgr, Lyn and Yrk, all of which are characterized by significant homology [49].

SFK are central mediators of proliferation, differentiation, migration, adhesion, invasion, and angiogenesis, and upon stimulation by growth factors, hormones, integrins or other factors, are activated throughout phosphorylation of signaling proteins for which the Src SH2 domain has high affinity.

SFK substrates include multiple downstream signaling pathway regulating cell proliferation, survival, motility, migration, cell-matrix adhesion dynamics, and regulation of cytoskeleton. Thus, not surprisingly, SFK play a significant role in signaling pathways involved in oncogenesis and tumor progression [50].

c-Src was the first proto-oncogene to be identified and an aberrant SFK activity has been reported in different tumor types, including breast, lung, colorectal cancer and pancreatic adenocarcinoma [51]. In most cases the overactivation of Src does not seem related to its mutations, but rather with overexpression and mutation of growth factor receptors or of other factors whose signaling pathways pass through SFKs.

Therefore SFKs have become a potentially attractive therapeutic target for different tumor types, and a number of SFKs are under evaluation in clinical trials.

Dasatinib (Sprycel^®^, Bristol-Myers Squibb), Saracatinib (Astra Zeneca), and Bosutinib (Bosulif, Pfizer) are all dual-specific inhibitors of SFKs and Abl and have been clinically tested mainly in trials of patients with haematological malignancies. Dasatinib and Bosutinib have both been approved by the FDA as second-line treatment for haematological malignancies.

These compounds have been tested in preclinical studies on xenograft mouse models of solid tumors with promising results [52]. However, the efficacy of SFKs as single agents has not yet been replicated in clinical trials of patients with solid cancers [53,54]. It therefore seems that SFK inhibitors may be more useful as part of combined treatments, also because SFKs are frequently activated in response to other treatments such as chemotherapy [55]. On the other hand, it is possible that the concomitant inhibition of c-Abl should be avoided in some cancer types [56], but there are few SFK inhibitors in preclinical development that are not also inhibitors of c-Abl.

As far as regards digestive endocrine tumors, the overexpression of Lck, a member of SFKs, has been demonstrated in metastatic progressive PETs, both at the RNA and protein levels [57]. The expression of LCK as detected by innunohistochemistry was more frequent in liver metastases than pancreatic primary lesions (66% *vs.* 43%), and was not limited to the plasma membrane, as in normal pancreatic islets, but often cytoplasmic, possibly due to overproduction of the protein, or to mutations that might impair palmitoylation or myristoylation which are critical for its localization.

The expression and activity of Src and Fyn and high levels of Src family activity in comparison to several cancer cell lines of different origins have been described in PET cell lines. Immunoreactivity for Src was also present in PET human samples. The inhibition of SFK activity in PET cell lines has been shown to interfere with adhesion, spreading and migration of such cells [58]. Known substrates of Src, such as p130Cas and FAK, were identified in this PET model.

More recently the possible link between the SFK and mTOR pathways in PETs has been investigated [36]. The findings suggest a novel role for SFKs in controlling mTOR activity during adhesion in PET cell lines. SFKs control mTOR activation at the periphery of the cells and regulate its translation of a subset of mRNA involved in cell cycle progression. Interestingly, we also found that concomitant inhibition of SFK and mTOR activities, strongly impaired PET cell line growth, compared to the effect exerted by the single agents.

Moreover, while the treatment of PET cells with mTOR inhibitor triggered a prosurvival response dependent on PI3K/AKT signaling in PET cells, as also shown in other types of cancer, the simultaneous inhibition of SFKs blocked this escape signal [33].

Thus, the link between SFK and mTOR in PET cells represents a potential target for combined treatment for PETs.

The relevance of SFKs in neuroendocrine tumors has also been underlined by the recent finding that neuroendocrine cancer stem cells (N-CSC), present strong activity of both the Src and mTOR pathways, and that targeting Src inhibits the growth both of N-CSCs cells *in vitro*, and of tumors derived from them *in vivo* [59].

Further studies employing SFK inhibitors, as single agents or in combination with mTOR inhibitors or other treatments, in animal models of GEP ETs could further support the relevance of SFKs in this tumor type, and eventually set the ground for clinical trials.

## 5. Prospect for Hedgehog Inhibitors

The hedgehog (Hh) pathway, initially discovered in *Drosophila*, is a major regulator for cell differentiation, tissue polarity and cell proliferation [60]. The seven transmembrane domain containing protein smoothened, frizzled family receptor (SMO), serves as the key player for the Hh pathway, whose function is inhibited by another transmembrane protein Patched (PTC) in the absence of Hh ligands [61]. Binding of Hh to its receptor PTC releases this inhibition, allowing SMO to signal downstream, eventually to glioma-associated oncogene family zinc finger (Gli) transcription factors, primarily Gli2 transcriptional factor. As transcription factors, Gli molecules can regulate target gene expression by direct association with a specific consensus sequence located in the promoter region of the target genes [62]. Previous studies had shown that the Hedgehog signalling pathway was aberrantly re-activated in several cancers arising from the gastrointestinal tract, including the majority of pancreatic cancers [63–67].

In a preclinical study, it was shown for the first time that the Hedgehog signaling pathway is expressed in the Rip1Tag2 mouse model of pancreatic islet carcinogenesis [68]. It was found that hedgehog blockade with cyclopamine, a natural chemical that belongs to the group of steroidal jerveratrum alkaloids, led to marked *in vivo* growth inhibition of islet cell tumors combined with a decrease in proliferating tumor cells and an augmentation of apoptosis. The downregulation of the Hedgehog target gene Gli1 was demonstrated in the tumor of cyclopamine treated mice, and a significantly prolonged survival of the cyclopamine treated Rip1tag2 mice *in vivo* was achieved. Cyclopamine has both teratogenic and antitumor activities arising from its ability to specifically block cellular responses to Hedgehog signaling by direct binding to Smoothened [69].

After proving the impact of cyclopamin on islet cell tumors in a transgenic mouse model of PETs the orally bioavailable smoothened antagonist LDE225 (Novartis Pharmaceuticals) was used [70]. Treatment with LDE225 reduces tumor volume by 95% in Rip1Tag2 mice. After treatment for several weeks, no invasive carcinoma was found in histopathological evaluation. Down regulation of the hedgehog target genes Gli1, Ptch1 and Hip was found in the tumor cells of LDE225 treated mice, and survival was prolonged in the LDE225 treated Rip1Tag2 mice. Thus, these results provided the first evidence that targeting the hedgehog pathway with the orally bioavailable Smo antagonist LDE225 may be a very attractive target for patients with PETs. Clinical evaluation of pharmacological Hedgehog blockade as a novel cancer treatment strategy has been hampered by the lack of suitable substances that might serve as future drugs for use in humans. The novel small molecule hedgehog pathway inhibitor LDE225 was designed with the intention to overcome this shortcoming. LDE225 is highly bound to human plasma proteins (>99%) and is readily water soluble [70].

In contrast to the negative influence of sonic hedgehog (Shh) on developing pancreatic growth, there does appear to be a role for Indian hedgehog (Ihh) in the adult pancreas. Ihh is expressed in islet and β-cells in characteristic, small, highly localized aggregates or punctuates [71–74]. Patched homolog protein (Ptc-1) and Smo have been shown to be expressed in islets, localized to β-cells by coexpression with insulin [71]. The insulin-secreting clonal cell line INS-1 expresses Ihh, Ptc-1, and Smo. Activation of the Hh signaling pathway by ectopic misexpression of Shh, increased activity of the rat insulin I promoter [72]. The administration of cyclopamine decreased insulin I promoter activity, decreased insulin secretion, and the insulin content of these cells in a concentration-dependent manner. It is well known, that the hedgehog pathway is activated in islet and β-cells [68,70]. Activation of the hedgehog signaling pathway by ectopic overexpression of Sonic hedgehog increased activity of the rat insulin I promoter. Therefore treatment with a hedgehog inhibitor might be of potential interest for patients with metastatic insulinoma. The effect of LDE225 was analysed to determine whether reduction of Hh signaling by LDE225 affects β-cell function *in vivo*. A quantitative real-time PCR was performed for treated and untreated Rip1Tag2 mice and it was found that inhibition of hedgehog signaling with LDE225 reduced endogenous insulin mRNA expression. Therefore, if LDE225 or other hedgehog inhibiting compounds are to be used in patients, attention should be given to changes in blood glucose levels [75].

In conclusion, targeting the hedgehog signaling pathway with LDE225 might be an interesting tool for future clinical testing in patients with PETs. Combination of LDE225 with already established drug regimens in PETs are likewise imaginable.

## 6. Conclusion

As the knowledge of molecular alterations in GEP ETs increases, a number of potentially “targetable” genes are being investigated. Among potential therapeutic strategies, the inhibition of angiogenesis and of the mTOR pathway, which are the better investigated targets, with approved drugs, still need investigation to elucidate the possible advantage of combined treatments in overcoming induced resistances, and in identifying biomarkers able to predict clinical efficacy, possibly employing biomarkers of response to treatment [76].

Other novel, interesting targets for which a strong biological rationale exists, and where specific inhibitors are available, are the Src Family Kinases and the Hedgehog Pathway.

A number of other specific critical issues which emerge should be carefully considered when designing clinical trials with targeted agents:

1. More preclinical studies on cell lines and animal models are needed to provide a stronger preclinical background in this field.
2. Only a minority of the drugs showing promising preclinical results have been tested in clinical trials (see Table 1).
3. There are no clinical trials specifically comparing one targeted therapy with another (*i.e.*, Everolimus *vs.* Sunitinib), or evaluating which sequence of treatments yields better results. This lack of knowledge does not permit the establishment of an evidence therapeutic algorithm for these patients.
4. There are few studies published up to now on evaluation of the combination of different targeted agents, with several others now still in the process of recruiting patients.

In conclusion, while there exists optimism, as novel therapeutic strategies are emerging for patients with GEP ETs, the increasing number of options also requires an increased need for accurate and rigorous studies to answer the clinically relevant questions.

## Figures and Tables

**Table 1:** Summary of clinical data on targeted agents for which a biological/preclinical rationale for the treatment of GEP ETs exists.

Compound	Target(s)	Published Preclinical studies (References)	Published Clinical Trials (References)	Approval	Ongoing Clinical Trials (Identifier)	Combination(s) Already Tested in Clinical Trials
Everolimus	mTOR	Yes [34,35,37,38]	Yes [34–42]	Yes (PETs)	Yes (NCT00688623 NCT01524783)	Yes (somatostatin analogues)
Sunitinib	VEGFR (1-2-3) PDGFRα, c-kit, Flt-3, RET	Yes [16–19]	Yes [22–24]	Yes (PETs)	Yes (SUNLAND STUDY)	No
Bevacizumab	VEGF	Yes [17]	Yes [26,27]	No	Yes (NCT00609765)	Yes (temozolamide, somatostatin analogues)
Brivanib	FGF, VEGF	Yes [29]	No	No	No	No
Cabozantinib	MET, VEGFR2	Yes [30]	No	No	Yes (NCT01466036)	No
BEZ235	Pan-I-PI3K	Yes [45]	No	No	Yes (NCT01658436)	No
Sorafenib	VEGFR2, PDGFR, FGFR1, FLT3	Yes [17–19,22]	No	No	Yes (NCT00942682)	No
Erlotinib	EGFR	Yes [77]	No	No	Yes (NCT00947167)	No
Vatalanib	VEGFR2, c-kit, PDGFR	Yes [17–19,22]	No	No	Yes (NCT00590343)	No
Src Inhibitors	SFKs	Yes [36,58]	No	No	No	No
LDE225	Hedgehog	Yes [63–67]	No	No	No	No

PETs = pancreatic endocrine tumors, SFKs = Src Family Kinases.
